# Hypothalamus-Anchored Resting Brain Network Changes before and after Sertraline Treatment in Major Depression

**DOI:** 10.1155/2014/915026

**Published:** 2014-03-20

**Authors:** Rui Yang, Hongbo Zhang, Xiaoping Wu, Junle Yang, Mingyue Ma, Yanjun Gao, Hongsheng Liu, Shengbin Li

**Affiliations:** ^1^The Affiliated Xi'an Central Hospital of Medical College of Xi'an Jiaotong University, Xi'an 710003, China; ^2^Key Laboratory of Environment and Gene Related Diseases, Ministry of Education, Xi'an Jiaotong University, Xi'an 710061, China; ^3^Xi'an Central Hospital, Xi'an 710003, China; ^4^Key Laboratory of Health Ministry for Forensic Science, Xi'an Jiaotong University, Xi'an 710061, China

## Abstract

Sertraline, one of the oldest antidepressants, remains to be the most efficacious treatment for depression. However, major depression disorder (MDD) is characterized by altered emotion processing and deficits in cognitive control. In cognitive interference tasks, patients with MDD have shown excessive hypothalamus activity. The purpose of this study was to examine the effects of antidepressant treatment (sertraline) on hypothalamus-anchored resting brain circuitry. Functional magnetic resonance imaging was conducted on depressed patients *(n=12)* both before and after antidepressant treatment. After eight weeks of antidepressant treatment, patients with depression showed significantly increased connectivity between the hypothalamus and dorsolateral prefrontal cortex, orbitofrontal cortex, anterior cingulate cortex, insula, putamen, caudate, and claustrum. By contrast, decreased connectivity of the hypothalamus-related areas was primarily located in the inferior frontal gyrus, medial frontal gyrus, cingulated gyrus, precuneus, thalamus, and cerebellum. After eight weeks of antidepressant therapy, 8 out of the 12 depressed subjects achieved 70% reduction or better in depressive symptoms, as measured on the Hamilton depression rating scale. Our findings may infer that antidepressant treatment can alter the functional connectivity of the hypothalamus resting brain to achieve its therapeutic effect.

## 1. Introduction

Depression is the commonest psychiatric disorder. It is the most disabling medical condition, in terms of years lost to disability, and it is predicted that depression will be the foremost contributor to the worldwide burden of disease by 2030 [1]. Depression is characterized by a profoundly negative view of the world, oneself, and the future [2], and this negative world view has been associated with negative biases in attention, interpretation, and memory [3]. In clinical settings, the depression is often misdiagnosed especially without a history of mania [4], leading to inadequate treatment, increased medical costs, and poor outcomes [5–7]. A recent upsurge of interest has been directed toward developing both diagnostic and prognostic biomarkers that can aid to diagnose which individuals are relatively more likely to progress clinically.

Previous studies using structural MRI have already revealed abnormalities in various brain areas and significant changes in the amygdala volume in adults suffering from depression and mood disorders [8]. The affective network largely consists of the prefrontal cortex as well as subcortical and allocortical brain structures such as the thalamus, amygdala, basal ganglia, and hippocampus. Studies probing this system demonstrated reduced frontal-subcortical connectivity in depression [9], which is thought to reflect impaired cognitive regulation of mood. Therefore, in particular functional MRI (fMRI), studies of the neural mechanisms of depression have shifted from those highlighting focal regions of abnormal brain functions to those focusing on the dysfunctional brain connectivity between spatially distinct brain regions. Resting state fMRI reflects the neuronal baseline activity of the brain, representing the state of the human brain without goal-directed neuronal action and external input [10], and the resting state functional connectivity in the blood oxygenation level-dependent signal during rest corresponds to consistent functionally relevant resting state networks (RSNs) [11]. With the benefit of its easy control and good acceptance, the resting state fMRI has received increasing attention for studying unipolar depression [12]. Some previous studies have observed increased connectivity within the default mode (DMN) network in depressed patients, and, interestingly, heightened DMN connectivity is also associated with depressive rumination [13, 14].

The hypothalamus is a subcortical region with roughly 4 cm^3^ neuronal tissue in human brain [15] located at the third ventricle, rostroventral to the thalamus. It consisted of several subareas embedded cytoarchitectonically distinguishable and proved to be functionally distinct with some functional overlaps [16]. Previous studies have demonstrated the involvement of hypothalamic nuclei in wide range of tasks including cognitive, behavioural, and visceral processes [17–21]. The hypothalamo-pituitary-adrenal (HPA) system constructs a common pathway in the mediation of the stress response. Cortisol normally exerts a negative feedback to shut down the stress response when facing the threat, acting upon the levels of the pituitary and hypothalamus. In addition, stimulation by corticosteroids can be exerted at the level of the amygdala, the prefrontal cortex, and the brain stem (locus coeruleus), interfering with HPA activity and stress effects on memory [22–24]. A large part of the environmental and genetic risk factors for depression appear to correlate with increased HPA-axis activity in adulthood. When patients or animals in models for depression are treated with antidepressants and electroconvulsive therapy or when patients show spontaneous remission, the HPA-axis function returns to normal [25]. Although antidepressant agents have been largely used for the treatment of major depressive disorder (MDD), the neurobiological mechanisms of their efficacy remains poorly understood [26–30]. Moreover, patients often present different clinical results: patients with fewer depressive episodes can achieve a rapid remission while others present with prolonged, remittent, or refractory illness [31–34]. In this respect, evaluating the brain functional antidepressant effects will allow a better understanding of the pathogenesis of MDD and its response to antidepressant treatment.

In the present study, we aimed to explore the effects of antidepressant treatment (sertraline) on functional connectivity network in unipolar depression while using the hypothalamus as the seed region. To address this question, we conducted a functional magnetic resonance imaging study of depression patients both before and after antidepressant treatment while they performed a resting scanning. We hypothesized that depression patients after medication would present increased, relative to their premedicated state.

## 2. Materials and Methods

### 2.1. Patients

Twelve major depression patients (7 males) participated in this study. This study was conducted at Xi'an Central Hospital. Each subject underwent a screening evaluation involving structured clinical interviews and assessments by trained clinicians and semistructured medical and psychiatric interviews with the study psychiatrist (K.L.P.). All subjects were characterized with the (1) structured clinical interview for DSM-IV; (2) Hamilton depression rating scale (HDRS).  Table 1 details the demographic and clinical characteristics of the subjects. All subjects had no history of head injury, organic mental disorders, alcohol or drug abuse, serious physical illness, or other mental illness. During the treatment, they were free of other treatments with psychiatric medications. None of the females were currently pregnant or lactating. All subjects provided written informed consent, and the study was approved by both local hospital institutional review boards. Head structural magnetic resonance imaging (MRI) results were evaluated by a single neuroimaging physician who was blinded to the experimental groups, and no obvious structural abnormalities were found.

### 2.2. Sertraline Treatment

Treatment consisted of the sertraline in a fixed-dosing design over 8 weeks (50–100 mg/day). Patients were evaluated at weeks 1 and 8 by the study psychiatrist (K.L.P.) in medication management sessions to assess symptom change and adverse events, with the target dose of 50–100 mg/day; clinical response was measured with the HDRS. Although we did not measure sertraline blood levels, the study psychiatrist and staff inquired about missed doses and conducted a pill count to confirm the subject report. No subject in the study ever missed more than 2 consecutive daily doses over the course of the 8-week study, and no subject regularly (>3 times) missed the dose.

### 2.3. Data Acquisition

The MR images were acquired with a 1.5 Tesla GE Excite MRI system (GE Health care Milwaukee, WI, USA). A foam pillow and a band across the forehead were used to fix the head. Resting state functional images were acquired with a single shot gradient recalled echo planar imaging sequence. The sequence covered the whole brain, axial view, parallel to the to the AC-PC line collected to cover the whole brain.

In AC-PC line, TR = 2500 ms, TE = 35 ms, resolution = 64 × 64, field of view (FOV) = 240 mm × 240 mm, flip angle = 90°, slice thickness = 3 mm without gap, 36 slices. A set of T1-weighted high-resolution structural images were collected using a 3D Fast SPGR sequence for anatomical localization. TR = 10.6 ms, TE = 4.8 ms, field of view (FOV) = 256 mm × 256 mm, flip angle = 15°, in-plane resolution = 1 mm × 1 mm, slice thickness: 1 mm without gap, 128 slices. All subjects were asked to remain relaxed without being engaged in any mental tasks. They were also instructed to keep their eyes closed but not fell asleep.

### 2.4. Image Preprocessing

All preprocessing steps were carried out using statistical parametric mapping (SPM5, http://www.fil.ion.ucl.ac.uk/spm/). Functional images were preprocessed using sinc interpolation for slice scan time correction, trilinear sinc interpolation for alignment (motion correction) of functional volumes, and high-pass temporal filtering to 1 Hz to remove slow drifts in the data. The image data were further processed with spatial normalization based on the MNI space and resampled at 2 mm × 2 mm × 2 mm. Resting data were also filtered using a band pass filter (0.01*∼*0.08 Hz) to reduce low-frequency drift and high-frequency noise. Finally, the functional images were spatially smoothed with a 6 mm full width at half maximum (FWHM) Gaussian kernel. All resting state functional images were preprocessed using statistical parametric mapping 5 (SPM5) and included motion correction, normalization, and smoothing.

### 2.5. Functional Connectivity Analysis

For each subject, the “seeding” time courses of the hypothalamus were, respectively, cross-correlated with all low-pass filtered voxels to generate functional connectivity maps within each of the three conditions. The resulting correlation coefficient t-maps were normalized and corrected to roughly standard normal distributions using methods previously described. The normality of the distribution was then tested using Kurtosis tests (*P* < 0.001, corrected). The maps of each individual were entered into one-sample *t*-tests, respectively, to determine whether group data was significantly different from zero. For visualization, all connectivity results were transformed into the Talairach stereotactic space and overlaid on MRIcro (http://www.mccauslandcenter.sc.edu/CRNL/) for presentation purposes. All resulting t-maps were then cluster-filtered to remove correlations involving less than three contiguous voxels and then superimposed on high-resolution anatomical images using a *P* < 0.001 cut-off threshold (FDR, corrected).

## 3. Results

Due to excessive head movement during scanning, imaging data from 2 patients were excluded and consequently a total of 10 participants were included in this study. Their demographic data and psychological scores are listed in Table 1. After eight weeks of antidepressant therapy, 8 out of the 12 depressed subjects achieved 70% reduction or better in depressive symptoms, as measured on the HDRS scale. Seven showed 50–70% improvement, and the remaining four showed 59–68% reductions. Thus, in our sample, approximately two-third of the sample showed very significant improvements in symptoms, and all patients responded at least partially to the treatment.

**Figure 1:**
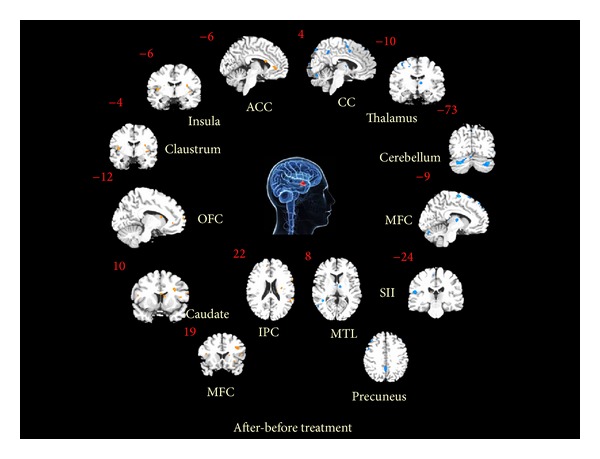
Both the increased and decreased hypothalamus-related functional connectivity resting brain networks after 8-week sertraline treatment for major depression (*P* < 0.01, FDR corrected).

Our results show that sertraline treatment has a larger impact on the hypothalamus-related brain networks for major depression patients (shown in Figure 1 and Table 2, *P* < 0.005, FWE corrected). After treatment, the hypothalamus showed prominently enhanced functional connectivity with the frontal cortex (mainly located in the dorsolateral prefrontal cortex, DLPFC; orbitofrontal cortex, OFC; superior frontal gyrus, SFG; and precentral gyrus), limbic system (anterior cingulate cortex, ACC; hippocampus), subcortical areas (insula, putamen, caudate, and claustrum), temporal lobe (superior temporal gyrus, STG), parietal (inferior parietal lobule, IPL; supramarginal gyrus, SG).

By contrast, sertraline treatment can also attenuate functional connectivity anchored by the hypothalamus for major depression patients. These areas were mainly located in the frontal cortex (inferior frontal gyrus, IFG; medial frontal gyrus, MFG; and superior frontal gyrus, SFG), limbic system (cingulated gyrus), temporal cortex (middle temporal gyrus, MTG), parietal cortex (precuneus), subcortical area (thalamus), and cerebellum (declive and uvula).

## 4. Discussion

The major finding of this study is that sertraline treatment exhibited larger impacts on the modulation of the hypothalamus-related functional connectivity brain network for major depression patients. These associations may be partially verified by the promotion of depression degree. As expected, depression degree showed significant differences after sertraline treatment. Our findings contribute to the growing evidence that sertraline treatment may be a beneficial effect on the depression patients and it mainly involved prefrontal-limbic-hypothalamus pathways. To our knowledge, this is the first in vivo magnetic resonance imaging study to demonstrate sertraline treatment impact on the hypothalamus-related resting brain network in depression patients.

Major depressive disorder is characterized by disruptions in executive control, linked to abnormal DLPFC function. The DLPFC plays an important role in working memory and other aspects of executive function [35, 36]. Previous studies have demonstrated structural changes in the prefrontal regions in major depressive disorder subjects, including decreases in cortical thickness and neural size, together with reductions in neural and glial density [37]. In the study by Thomas et al., ischemia in the white matter of DLPFC was found in subjects with late-life depression [38] and lends support to the “vascular depression” hypothesis [39, 40]. Results from these studies associate depression with abnormally low levels of DLPFC activity [41, 42]. Our findings were consistent with studies linking the DLPFC with depression and further proved that sertraline treatment can effectively improve the functional connectivity between hypothalamus and DLPFC.

The major physiological response to stress involves activations of neuroendocrine systems, mainly through the hypothalamus-pituitary-adrenal (HPA) axis.

Emotional stimuli always reach the HPA axis via the amygdala and descending pathways from the forebrain. The amygdala exerts excitatory control over the hypothalamus to stimulate the HPA axis, which, via increased cortisol levels, acts in a positive feedback manner to further stimulate the amygdala [43]. By contrast, the hippocampus exerts inhibitory control over the HPA axis, and hippocampus acts in a negative feedback manner to inhibit the HPA axis. This is crucial for limiting the activity of the HPA axis, since that, without it, the positive feedback loop via the amygdala would cause the system to run out of control [44–46]. Descending negative feedback over the HPA axis is also exerted at the level of the dorsomedial prefrontal cortex (PFC) and the prelimbic cortex [47] so activation of these areas by emotional self-regulation can also improve the control over the HPA axis. Our results were consistent with the inference that sertraline treatment can effectively improve the functional connectivity between the hypothalamus and hippocampus.

The functional role of the cingulate cortex—and specifically the ACC—in depression is well described [48]. Our data shows significant enhancement of functional connectivity between the hypothalamus and ACC. This region is also a key component of the default mode network [49]. Abnormal resting state functional connectivity of these same regions has been described in depression [13]. Previous work using DTI data shows that connectivity in the cingulate portion of the cingulate bundle has structural changes with depression at baseline and also has an impact on remission [50]. The OFC has extensive connections with the limbic system, including the cingulate cortex [51]. The presence of enhanced functional connectivity with the hypothalamus in both ACC and the OFC supports the notion that sertraline treatment can improve the damages on these regions.

Another intriguing research is the decreased functional connectivity between the hypothalamus and cerebellum (located in the posterior part). The cerebellum generally accepts the information from temporal lobe, prefrontal lobe, and cingulated gyrus and these areas have exerted controls on the cognitive and emotional regulation [52]. It is also emphasized that the involvement of the cerebellum in cognitive and emotional controls and proposed the concept of the cerebellar cognitive affective syndrome, which usually refers from the affective bluntness and depression to affective disorder, and finally appears in execution, visual, spatial and language dysfunction [53]. Our results proved that the hypothalamus may project attenuated influence on the cerebellum after the sertraline treatment.

Limitations of this study include potential bias given that only small samples were included in the current study. Further study will include much larger sample patients and verify our hypothesis. Other factors not included in this analysis, such as age of onset, illness duration, and prior antidepressant use, may influence the hypothalamus-related resting brain network in depression. In addition, and perhaps most critical, was a lack of data on medication history. Since the majority of subjects had long-term depression (average episode duration = 4.04 years), they were likely to have taken antidepressants prior to study baseline; this prior medication use would likely have had a greater influence on functional brain networks than would concurrent medications, if any relationship exists. These factors should be addressed in future studies.

## Figures and Tables

**Table 1:** Demographic and clinical characteristics of the subjects.

	Depression group	
	Mean	SD
Age (years)	34.91	12.16
Duration (years)	4.04	3.14
Height (cm)	168.3	6.54
Weight (kg)	79.51	9.96
Hamilton depression (before)	24.08	4.40
Hamilton depression (after)	5.83	2.32

**Table:** (a) Increased

		Talairach			*t* value	Voxels
		*x*	*y*	*z*		
Frontal						
DLPFC BA/44	R	48	16	10	3.08	44
OFC BA/10	R	8	38	−7	2.25	44
SFG BA/8	R	14	39	48	2.33	22
Precentral gyrus BA/44	R	44	12	9	3.20	20
Limbic						
ACC BA/24	R	6	31	0	2.76	45
Hippocampus	R	33	−14	−18	3.23	16
Subcortical						
Insula BA/13	R	42	12	10	2.61	43
Putamen	R	24	8	−2	2.09	28
Caudate	R	8	10	11	3.57	82
Claustrum	R	32	−3	13	2.54	25
Temporal						
STG BA/22	R	63	−40	20	2.37	27
Parietal						
IPL BA/40	R	55	−44	22	2.40	38
SG BA/40	R	57	−37	30	2.33	24

**Table:** (b) Decreased

		Talairach			*t* value	Voxels
		*x*	*y*	*z*		
Frontal						
IFG BA 47	L	53	17	−6	−2.87	54
IFG BA 9	R	−53	11	31	−2.35	427
Precentral gyrus	L	−49	−8	43	−2.29	16
MFC BA 6/8	R	6	−1	61	−2.98	144
SFG BA 6	L	−2	28	54	−2.86	112
	R	8	−1	63	−3.04	152
Limbic						
Cingulate gyrus	L	−6	6	40	−2.63	155
BA 32	R	4	10	40	−2.32	88
Temporal cortex	L					
MTL BA/37	R	−53	−66	7	−2.48	48
Parietal cortex						
Precuneus	L	−4	−73	50	−2.68	137
BA 7	R	4	−44	45	−2.19	37
Subcortical						
Thalamus	L	−4	−4	4	−2.34	30
	R	8	−9	8	−2.75	28
Cerebellum						
Declive	L	−16	−82	−16	−3.46	455
	R	36	−75	−21	−3.07	290
Uvula	L	−28	−71	−23	−3.19	111
	R	34	−63	−25	−3.24	110

Abbreviations: BA: Brodmann area; DLPFC: dorsolateral prefrontal cortex; OFC: orbitofrontal cortex; SFG: superior frontal gyrus; ACC: anterior cingulate cortex; STG: superior temporal gyrus; IPL: inferior parietal lobule; SG: supramarginal gyrus; IFG: inferior frontal gyrus; MFC: medial frontal cortex; MTL: medial temporal lobule.
